# ARF GTPases and Their Ubiquitous Role in Intracellular Trafficking Beyond the Golgi

**DOI:** 10.3389/fcell.2021.679046

**Published:** 2021-07-22

**Authors:** Petia Adarska, Luis Wong-Dilworth, Francesca Bottanelli

**Affiliations:** Institut für Biochemie, Freie Universität Berlin, Berlin, Germany

**Keywords:** membrane trafficking, Golgi, adaptors, ARF GTPase, TGN, endosomes, clathrin, COPI

## Abstract

Molecular switches of the ADP-ribosylation factor (ARF) GTPase family coordinate intracellular trafficking at all sorting stations along the secretory pathway, from the ER-Golgi-intermediate compartment (ERGIC) to the plasma membrane (PM). Their GDP-GTP switch is essential to trigger numerous processes, including membrane deformation, cargo sorting and recruitment of downstream coat proteins and effectors, such as lipid modifying enzymes. While ARFs (in particular ARF1) had mainly been studied in the context of coat protein recruitment at the Golgi, COPI/clathrin-independent roles have emerged in the last decade. Here we review the roles of human ARF1-5 GTPases in cellular trafficking with a particular emphasis on their roles in post-Golgi secretory trafficking and in sorting in the endo-lysosomal system.

## Introduction

ADP-ribosylation factor GTPases are major regulators of intracellular trafficking. Based on sequence similarities, they have been classified into type I (ARF1-3; humans lacking ARF2), type II (ARF4 and ARF5) and type III with the only member being the PM-localized ARF6. ARFs on/off switch is tightly regulated by guanine nucleotide exchange factors (GEFs), that turn ARFs on and GTPase activating proteins (GAPs), that trigger GTP hydrolysis, returning ARFs to the off state (Sztul et al., 2019). Hence, GEFs and GAPs establish the duration of signaling driven by specific ARFs. Activation of ARFs by GEFs triggers exposure of a N-terminal amphipathic helix that is important for membrane association and has an active role in membrane curvature generation (Beck et al., 2008). GTP binding and the downstream conformational changes in ARFs are crucial for effector recruitment and to trigger a specific biological response. Importantly, while numerous ARF GEFs and GAPs have been identified, not much is known about which GEF/GAP/ARF coordinates which specific cellular function (for a more in-depth review regarding the function and localization of the ARF GEFs and GAPs see Sztul et al., 2019). Intriguingly, although the ARF-GTP conformation is referred to as the “active” state, ARF proteins can also interact with effectors in the “inactive” ARF-GDP bound state. This is the case of ARF6-GDP, which engages with regulators of other small G proteins, raising the interesting possibility that GDP and GTP bound ARF6 would lead to activation of alternate signaling pathways depending on the bound nucleotide [reviewed in Donaldson and Jackson (2011)]. Turning off ARFs is also crucial for their function as failure to inactivate ARFs can block downstream trafficking. For example, a GTP-locked ARF6 mutant blocked membrane recycling between the PM and endosomes (Eyster et al., 2009) and the GTP-locked form of ARF1 prevented cargo loading into COPI vesicles (Malsam et al., 1999), highlighting the importance of GTP hydrolysis beyond being a mere turning off switch.

ADP-ribosylation factors are highly conserved in sequence and structure, with type I ARFs >96% identical, type II ARFs 90% identical to each other and 80% identical to type I ARFs and ARF6 >65% identical to type I and II ARFs. ARFs possess two switch regions (switch I and II), which change conformation upon GTP binding and mediate the interaction with effectors and regulators. In all ARF family members, switch I and II are almost identical (Goldberg, 1998; Pasqualato et al., 2001). Due to the very high sequence and structural similarities, it is still unclear what drives ARFs association with different intracellular membranes and how effectors are differentially recruited to trigger a specific cellular response. Interestingly, GTP-bound, but not GDP-bound, ARF1 and ARF6 are structurally very similar, suggesting ARFs may discriminate between effectors in their inactive form (e.g., binding to different GEFs for activation). The structures of GDP-bound ARF6 and ARF1 show that differences in sequence outside of the switch regions result in conformational differences within the switch regions, possibly driving the specificity for regulators in living cells (Menetrey et al., 2000). While structural differences have been highlighted for ARF1 and ARF6, which are the most divergent ARFs in sequence, structural data is not available for the other type I and II ARFs with a higher degree of sequence similarity. Recruitment of ARFs to intracellular membranes was also shown to be driven by the interaction of ARFs with specific membrane localized receptors. A specific 16 amino acid sequence in ARF1 is responsible for its recruitment to early Golgi cistearnae via the SNARE membrin (Honda et al., 2005). ARF1-GDP is additionally recruited to the Golgi via binding to p23, a member of the p24 family of transmembrane proteins via its C-terminus. The *trans*-Golgi network (TGN) localization of ARF3 is also determined by its C-terminus, making it unlikely that membrane association is driven by the interaction with the activating GEF, as the surface interacting with the Sec7 domain lies on the opposite side of the protein. This suggests the presence of a yet to be identified receptor for ARF3 recruitment to the TGN membranes (Manolea et al., 2010).

In terms of cellular functions, the role of ARF1 in the formation of COPI vesicles at the Golgi has been extensively studied through *in vitro* reconstitution or hybrid approaches using COPI budding assays in semi-permeabilized cells and purified Golgi membranes (Orci et al., 1986; Reinhard et al., 2003; Adolf et al., 2013). These studies led to a detailed molecular understanding of the ARF on/off switch. However, in the test tube, the specificity of action driven by the specific recruitment of a GTPase and its regulators to a sub-cellular membrane is lost. Studies of ARF function had initially been primarily focused on understanding how ARF1 regulates COPI coat recruitment. In the last decade, various publications have highlighted the roles of type I and II ARFs in post-Golgi trafficking steps, including exocytosis, endo-lysosomal trafficking and also coat-independent mechanisms of action. In this short review, we will focus on what is known about ARFs in terms of localization as well as function in post-Golgi secretory and endosomal trafficking.

## ARF Proteins Show Distinct as Well as Overlapping Distribution Throughout the Cell

**FIGURE 1:**
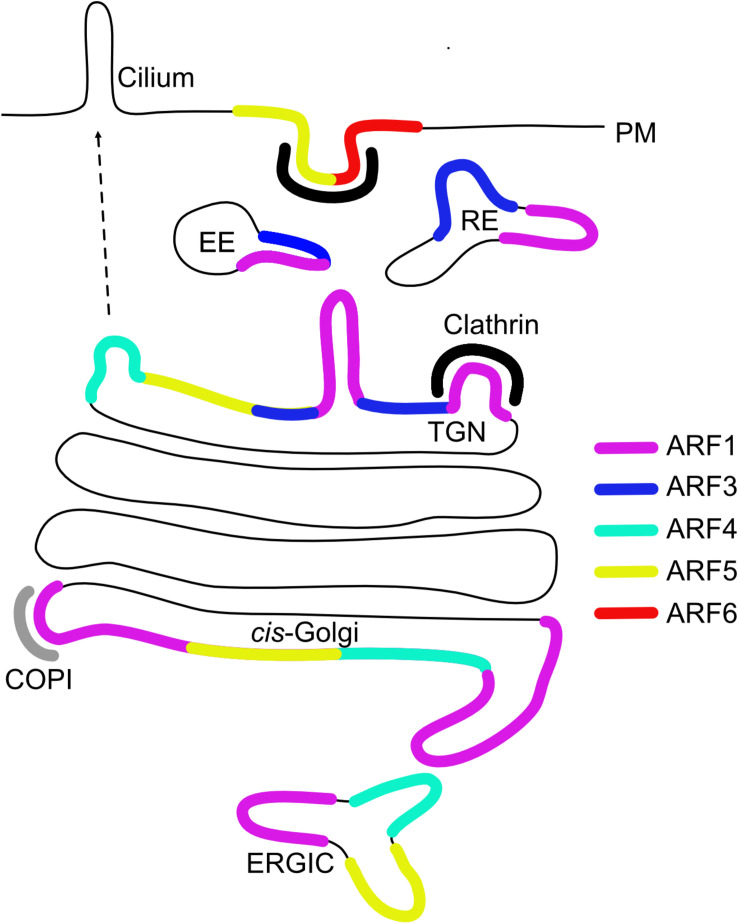
Intracellular localization of ARF GTPases. Early endosome (EE), recycling endosome (RE), *trans*-Golgi network (TGN), ER-Golgi intermediate compartment (ERGIC), and plasma membrane (PM).

Types I and II ARFs have all been localized to the Golgi apparatus, while the sole type III member ARF6 is the only non-Golgi associated ARF and localizes to the PM and endosomes (Figure 1; Sztul et al., 2019). When dissecting the intra-Golgi localization, ARF1, ARF4 and ARF5 localize to the *cis* cisternae and the TGN, whereas ARF3 localization is limited specifically to the TGN due to its two unique C-terminal determinants (A174 and K180), which are conserved among ARF3 homologs across species (Claude et al., 1999; Kawamoto et al., 2002; Deretic et al., 2005; Honda et al., 2005; Mazelova et al., 2009; Manolea et al., 2010; Sadakata et al., 2010). Golgi-associated ARFs are responsible for COPI recruitment, however, it is unclear which ARF contributes to COPI vesicles formation in living cells. The double knockdown (KD) of ARF1 and ARF4 was reported to trigger dissociation of COPI from the Golgi (Volpicelli-Daley et al., 2005) and ARF1, ARF4, and ARF5 all support COPI vesicle formation of *in vitro* generated vesicles from Golgi membranes (Popoff et al., 2011). Moreover, COPI vesicles generated in semi-permeabilized cells with type I or type II ARFs show similar content (Adolf et al., 2019).

Although predominantly at the Golgi, fluorescently tagged ARF1 and type II ARFs were also reported to localize to peripheral ERGICs in living cells (Chun et al., 2008). Tubulation of the ERGIC after simultaneous depletion of ARF1 and ARF4 already hinted to an involvement of both type I and type II ARFs in sorting at the ERGIC a few years earlier (Volpicelli-Daley et al., 2005). However, type I and II ARFs show differential membrane association dynamics, suggesting a functional difference (Duijsings et al., 2009).

GFP-tagged ARF1 and ARF3 have been shown to localize to recycling endosomal compartments containing endocytosed transferrin (Tfn) and their simultaneous depletion inhibited PM recycling of Tfn (Kondo et al., 2012). Furthermore, ARF1 and ARF3 have been found on Rab4-positive endosomal membranes (D’Souza et al., 2014).

At the PM and endosomes, ARF6 is involved in clathrin-mediated endocytosis, clathrin-independent endocytosis and recycling pathways (D’Souza-Schorey et al., 1995; Peters et al., 1995; Donaldson, 2003). Interestingly, while ARF6-GTP localization is restricted to clathrin coated pits and endocytic vesicles, its role may be downstream of endocytosis in facilitating fast PM recycling (Montagnac et al., 2011). Aside from its role at the Golgi, ARF5 has also been found to act at the PM. Here, ARF5 controlled integrin endocytosis and was localized to clathrin-coated pits together with IQSEC1/BRAG2, a GEF known for activating ARF6 (Moravec et al., 2012). Recently, ARF5 and IQSEC1 were also implicated in Ca^2+^-dependent disassembly of focal adhesions during cell migration (D’Souza et al., 2020). Further supporting a role for ARF5 in migration, the GTPase was shown to be recruited to vesicular structures in cell protrusions via IQSEC1 and to promote invasion and metastatic cancer by controlling phosphoinositide metabolism (Nacke et al., 2021).

In conclusion, ARFs show overlapping yet distinct distribution and functions throughout the secretory and endocytic pathways. This complexity makes the investigation of their diverse functions and the identification of specific regulators and effectors especially challenging. In particular, because of the high sequence similarity, the endogenous localization of all ARFs is not yet known and localization studies have relied on the overexpression of tagged GTPases until very recently. A promising technique that will help define the roles of ARFs in living cells is gene editing, which has been instrumental in highlighting the multiple roles of ARF1 at the Golgi (Bottanelli et al., 2017).

## The Role of ARFs in Adaptor Recruitment

At the TGN, cargoes are sorted into distinct classes of vesicles destined either to the endo-lysosomal system or to the PM for secretion. Interestingly, direct, as well as indirect, secretory pathways that detour via endosomal compartments have been characterized (Stalder and Gershlick, 2020). At the TGN and in downstream endosomal compartments, ARFs have a role in recruiting cargo adaptor proteins, such as the oligomeric adaptor protein (AP) complexes and the monomeric Golgi-localized, γ-ear-containing, ARF-binding proteins (GGAs).

AP1-4 are known to function at the TGN and/or downstream endosomes (Tan and Gleeson, 2019). Still, the exact cellular function of AP complexes is not fully understood - which in particular extends to their interaction with different ARFs. The best studied case is the ARF1-dependent recruitment of AP-1 to the TGN. TGN-associated GTP-bound ARF1 recruits cytosolic AP-1 and changes the conformation of AP1 from closed to open, thus “unlocking” AP-1 and allowing binding to the sorting motifs of cargoes (Stamnes and Rothman, 1993; Ren et al., 2013). Membrane curvature is then induced by AP interaction partners, such as coat (e.g., clathrin) and accessory proteins, initiating the formation of vesicles. The crystal structures in Ren et al. (2013) highlight the structural change of AP-1 upon ARF1 activation and demonstrate that ARF1 binds the β1 and γ subunits of AP-1 via its switch I and II motifs. However, the switch I and II residues are highly conserved among ARFs. Therefore, the observed recruitment of AP-1 to the membrane could be also driven by other ARFs, which have been localized to late Golgi compartments (like ARF3). Additionally, AP-3 and AP-4 are structurally similar to AP-1 (Ren et al., 2013) and have also been shown to be recruited to membranes via interaction with ARF1 (Ooi et al., 1998; Boehm et al., 2001). AP-3 has a role in transport to late endosomes (LEs) and lysosomes (Peden et al., 2004), while AP-4 has recently been implicated in autophagosomal membrane formation from the TGN (Mattera et al., 2017). It would be interesting to explore whether different AP complexes are recruited through different membrane-localized ARFs. A better understanding of the localization of ARFs in the endo-lysosomal system will be necessary to understand which ARF recruits which AP on a specific cellular membrane.

Monomeric adaptors of the GGA family are also recruited to the TGN by binding to ARFs via their GAT domain (Collins et al., 2003). Three human GGA proteins (GGA1-3) have been identified and have all been localized to the TGN, where it is unclear whether they have distinct or overlapping roles in the transport to the lysosome (Ghosh and Kornfeld, 2004). Interestingly, the TGN association of GGAs relies on coincidence detection via ARF1 and phosphatidylinositol-4-phosphate (PI4P) (Dell’Angelica et al., 2000; Puertollano et al., 2001; Wang et al., 2007; Kametaka et al., 2010), as for AP-1 (Wang et al., 2003). Thus, PI4P plays a general role in ensuring the specificity of recruitment of various machinery to the TGN, as adaptors and other ARF effectors, like four-phosphate-adaptor protein (FAPP), require the concomitant presence of phosphoinositide (PI) species and specific ARFs to associate with membranes. PI4P is predominantly enriched at the TGN and regulated by phosphatidylinositol-4-OH kinases (PI4Ks), specifically PI4KIIIβ and PI4KIIα (Makowski et al., 2020). It has been reported that ARF1 mediates PI4KIIIβ activation and recruitment to the Golgi membrane (Godi et al., 1999; Haynes et al., 2005), where PI4KIIIβ can then stimulate PI4P synthesis (Godi et al., 1999). Although this is an interesting model that puts ARF1 functionally upstream of other PI4P-binding proteins, there are also other proteins that may mediate PI4KIIIβ membrane association (Waugh, 2019). Additionally, membrane contact sites between the TGN and the ER have been shown to have a role in regulation of TGN PI4P levels (Mesmin et al., 2013; Capasso et al., 2017; Wakana et al., 2021).

In conclusion, various ARFs, together with PI4P, may provide the molecular specificity for cargo selection mediated by adaptors. While ARF1 is again the best studied member, investigating the role of the other ARFs at the TGN may bring some interesting insights.

## The Diverse Functions of ARFs at the TGN

ARF1 is responsible for the recruitment of the adaptor AP-1 and clathrin to the TGN. AP-1 has a role in diverse sorting steps, including constitutive secretion, basolateral sorting in polarized cells, as well as bi-directional communication between TGN and endosomes (Stamnes and Rothman, 1993; Folsch et al., 1999; Wang et al., 2003; Chi et al., 2008). It is unclear whether ARF1 is the sole player in the recruitment of AP-1 to different membranes. In fact, depletion of both ARF1 and ARF4 was necessary to trigger dissociation of AP-1 from Golgi membranes (Nakai et al., 2013). Whether ARF1 and ARF4 act in concert or have redundant functions remains to be investigated. The Golgi localization of ARF3 is confined to the TGN, making it a potential candidate for adaptor recruitment for post-Golgi trafficking. However, the high sequence similarity between the type I ARFs (ARF1 and ARF3) and the lack of known differential interactors has made it very hard to pin-point any functional differences between the two members. Interestingly, exit of secretory cargoes from the TGN is inhibited at 20°C and ARF3 dissociates from the membrane at this temperature. However, membrane dissociation of ARF3 does not seem to be the cause of the blockage of secretory trafficking as ARF3 KD does not affect AP-1 and PI4P levels (Manolea et al., 2010). The Brefeldin A-inhibited guanine nucleotide exchange factors 1 and 2 (BIG1 and BIG2) have been shown to activate both ARF1 and ARF3 for the downstream recruitment of AP-1, suggesting a role of ARF3, in addition to ARF1, in AP-1-dependent pathways (Lowery et al., 2013). Further, ARF3 regulates trafficking of Toll-like receptor 9 (TLR9) to endo-lysosomes, possibly by facilitating TGN export (Wu and Kuo, 2015). Even though ARF1, ARF3, and ARF4 have all been implicated in TGN export, it has been difficult to directly pin-point the exact place of action of these ARFs using functional trafficking assays. Diffraction-limited light microscopy does not provide sufficient resolution to clearly separate Golgi cisternae and ERGIC elements, making it hard to identify the exact place where trafficking is impaired upon ARF depletion. While single ARFs were shown not to have an effect on secretory trafficking, in particularly on TGN export of vesicular stomatitis virus G (VSV G) protein (Volpicelli-Daley et al., 2005), it was recently reported that KD of ARF1 and ARF4 inhibit TGN export of the β-secretase BACE1 but not amyloid precursor protein (APP) (Tan et al., 2020). The latter study suggests that specific ARFs may be required for the trafficking of specific cargoes via recruitment of different adaptors.

Another recently highlighted role for ARF1 is the formation of tubular vesicular trafficking intermediates containing secretory cargoes at the TGN. Interestingly, these tubules contain discrete patches of clathrin but it is unclear whether clathrin has a functional role or if adaptors are involved in this process (Bottanelli et al., 2017). ARF1 interacts with effectors, like the PH-domain containing protein FAPP, which may facilitate membrane deformation and aid tubulation (for more details, read Stalder and Gershlick, 2020). In addition, ARF1 tubules do not fuse with the PM, suggesting they either lose ARF1 prior to fusion or an intermediate sorting station may be present (Bottanelli et al., 2017).

ARFs also seem to possess specialized secretory functions in specific cell types. ARF4 and ARF5 have been shown to regulate biogenesis/trafficking of dense core vesicles (DCVs) in rat PC12 cells (Sadakata et al., 2010). DCVs are important carriers, which transport cargoes like neuropeptides to the nerve terminal in neurons. Interestingly, here GDP-bound ARFs are recruited to the TGN by calcium-dependent activator protein for secretion 1 (CAPS1). ARF4 was also shown to specifically bind the C-terminal ciliary target signal on rhodopsin for its transport to the primary cilium (Deretic et al., 2005) and the various GEFs and GAPs involved in ARF4-dependent ciliary transport have also been identified (Deretic et al., 2021).

In summary, all four type I and II ARFs are implicated in TGN export, AP-1 dependent pathways or secretion in specialized cells. However, it is unclear which ARF (if any) is responsible for constitutive secretion and direct TGN-to-PM pathways. Additionally, more specialized functions of ARFs in different cell types may have yet to be discovered.

## The Role of ARFs in Trafficking in the Endo-Lysosomal System

ARFs and their effectors are emerging as key components of the molecular machinery mediating cargo sorting in endosomal pathways. Recycling endosomes (REs) are involved in recycling of cargoes back to the PM after endocytosis and from endosomes back to the TGN. Volpicelli-Daley et al. (2005) were among the first to investigate the role of ARFs in endosomal recycling pathways. Their approach consisted of monitoring the morphology of endosomes, as well as various membrane trafficking steps upon depletion of human ARF1-5 in HeLa cells. As observed for other trafficking steps, KD of individual ARFs was not sufficient to alter the morphology of Tfn-positive REs. However, simultaneous KD of ARF1 and ARF3 caused REs tubulation (Volpicelli-Daley et al., 2005; Kondo et al., 2012) and inhibited endocytic recycling of the Tfn receptor (Volpicelli-Daley et al., 2005). This is in agreement with the observation that the ARF GEF BIG2 has a role in the maintenance of the structural identity of REs through activation of ARF1 and ARF3 (Shin et al., 2004). Additionally, ARF1 and ARF4 double KD also resulted in tubulation of REs and inhibited retrograde endosome-to-TGN transport (Nakai et al., 2013), without affecting endocytic recycling (Volpicelli-Daley et al., 2005; Nakai et al., 2013). ARF4 has been recently identified as an interactor of active GTP-bound Rab30, which also plays a role in the same recycling pathway (Zulkefli et al., 2021). Taken together, ARF1 may differentially regulate export from REs to either the PM (together with ARF3) or the TGN (with ARF4).

ARFs have also been implicated in sorting processes at early endosomes (EEs). EEs are sorting hubs that can either sort proteins to the PM, to the TGN or to lysosomes for degradation. Tubular domains on EEs are thought to be responsible for retrograde EE-to-TGN transport and cargo recruitment is mediated via various adaptors (GGAs, AP-1, and AP-3). D’Souza et al. (2014) reported that ARF1 KD, but not ARF3 KD, was sufficient to induce the formation of long tubules emanating from Rab4-positive EEs and to trigger membrane dissociation of AP-1 and AP-3. KD of either ARF1 or ARF3, however, resulted in dissociation of GGA3 from EEs, suggesting that ARF1 and ARF3 may have overlapping (GGA3 recruitment) yet distinct (AP-1 recruitment) sorting roles on EEs.

Regulators of ARF function have also been extensively studied and provide, albeit indirectly, proof of the importance of ARF-mediated sorting in the endosomal system. The GEFs BIG1 and BIG2 localize to the TGN and endosomes, where they activate ARFs resulting in recruitment of adaptors (Shinotsuka et al., 2002; Zhao et al., 2002; D’Souza et al., 2014). Interestingly, catalytically inactive BIG2 induces tubulation of REs (Shin et al., 2004), similar to what was observed when depleting BIG2 (but not BIG1) via small interfering RNA (Ishizaki et al., 2008). In particular, downregulation of BIG2 affected protein association with the REs, whereas simultaneous depletion of BIG1 and BIG2 resulted in the dissociation of TGN and RE proteins, as well as impaired retrograde trafficking from the LEs to the TGN (Ishizaki et al., 2008). This suggests a model in which BIG2 functions at the REs, while BIG1 and BIG2 may cooperate to regulate retrograde transport from LEs back to the TGN.

Next, various ARF GAPs have also been implicated in the regulation of ARFs beyond the Golgi. AGAP1 reportedly interacts with AP-3, regulating trafficking from the TGN to the lysosomes (Nie et al., 2003) and AGAP2 was shown to interact with AP-1 (Nie et al., 2005). A gene mutated in amyotrophic lateral sclerosis (ALS) and frontal temporal degeneration (FTD), called C9orf72, was recently identified as a GAP for ARF1. Intriguingly, C9orf72 localization is restricted to lysosomes under amino acid starvation conditions (Su et al., 2020) and has been suggested to regulate ARF1 in *trans* via interaction of lysosomes and ARF1-positive membranes.

Like ARFs themselves, their GEFs and GAPs may be recruited to multiple locations where they have different functions. Pinpointing the function of distinct ARFs, as well as their GEFs and GAPs is a challenging task, further complicated by the lack of a complete understanding of the sorting pathways emerging from the TGN. Additionally, it should be also considered that regulatory networks of the various ARFs may be closely interconnected via ARF cascades, explaining why multiple GTPases have been found to interact with the same ARF regulator (Stalder and Antonny, 2013).

## Outlook

Untangling the cellular ARF code will be a challenge due to the complexity of ARF synergies, their differential functions and the lack of knowledge about the specificity of each ARF for GAPs and GEFs *in vivo*. Thus, an interesting challenge ahead is to investigate the specific interplay between GEFs/GAPs and ARFs as well as their nanoscale localization in an unperturbed cellular environment. ARF GTPases are the master regulators of intracellular trafficking. While we know a lot about the molecular mechanisms governing their GTPase cycle, it has been incredibly hard to study their function in living cells due to their high sequence and structural similarity and possible functional redundancy. The fact that concomitant depletion of two ARFs is often necessary to observe a cellular phenotype raises the interesting possibility that ARFs may be acting in pairs. Work from the Weiland lab has shown that ARF1-GTP dimers form *in vitro* and may be responsible for the scission of vesicles (Beck et al., 2008, 2011). A recent publication shows that ARF-GEF dimers recruit two closely spaced ARF1-GTP to the membranes, promoting vesicles formation (Brumm et al., 2020), supporting a functional role for ARF dimers in living cells. Further work will be required to test whether different ARF heterodimers are responsible for selective recruitment of various downstream effectors and cargoes via differential adaptor recruitment.

## Author Contributions

All authors wrote the manuscript, contributed to the article, and approved the submitted version.

## Conflict of Interest

The authors declare that the research was conducted in the absence of any commercial or financial relationships that could be construed as a potential conflict of interest.
